# Reactive astrocytes undergo M1 microglia/macrohpages-induced necroptosis in spinal cord injury

**DOI:** 10.1186/s13024-016-0081-8

**Published:** 2016-02-03

**Authors:** Hong Fan, Kun Zhang, Lequn Shan, Fang Kuang, Kun Chen, Keqing Zhu, Heng Ma, Gong Ju, Ya-Zhou Wang

**Affiliations:** Department of Neurobiology and Collaborative Innovation Center for Brain Science, School of Basic Medicine, Fourth Military Medical University, Xi’an, 710032 China; Department of Orthopedics, Tangdu Hospital, Fourth Military Medical University, Xin Si Road, Xi’an, Shaanxi 710038 China; Zhejiang University China Brain Bank, Department of Pathology and Pathophysiology, Department of Neuroscience, 866 Yu-Hang-Tang Road, Zhejiang University Zi-Jin-Gang Campus, Hangzhou, Zhejiang 310058 China; Department of Physiology & Department of Pathophysiology, School of Basic Medical Sciences, Fourth Military Medical University, 169 Chang Le Xi Road, Xi’an, Shaanxi 710032 China

**Keywords:** Spinal cord injury, Reactive astrocytes, Necroptosis, M1 microglia/macrophage

## Abstract

**Background:**

A unique feature of the pathological change after spinal cord injury (SCI) is the progressive enlargement of lesion area, which usually results in cavity formation and is accompanied by reactive astrogliosis and chronic inflammation. Reactive astrocytes line the spinal cavity, walling off the lesion core from the normal spinal tissue, and are thought to play multiple important roles in SCI. The contribution of cell death, particularly the apoptosis of neurons and oligodendrocytes during the process of cavitation has been extensively studied. However, how reactive astrocytes are eliminated following SCI remains largely unclear.

**Results:**

By immunohistochemistry, *in vivo* propidium iodide (PI)-labeling and electron microscopic examination, here we reported that in mice, reactive astrocytes died by receptor-interacting protein 3 and mixed lineage kinase domain-like protein (RIP3/MLKL) mediated necroptosis, rather than apoptosis or autophagy. Inhibiting receptor-interacting protein 1 (RIP1) or depleting RIP3 not only significantly attenuated astrocyte death but also rescued the neurotrophic function of astrocytes. The astrocytic expression of necroptotic markers followed the polarization of M1 microglia/macrophages after SCI. Depleting M1 microglia/macrophages or transplantation of M1 macrophages could significantly reduce or increase the necroptosis of astrocytes. Further, the inflammatory responsive genes Toll-like receptor 4 (TLR4) and myeloid differentiation primary response gene 88 (MyD88) are induced in necroptotic astrocytes. *In vitro* antagonizing MyD88 in astrocytes could significantly alleviate the M1 microglia/macrophages-induced cell death. Finally, our data showed that in human, necroptotic markers and TLR4/MyD88 were co-expressed in astrocytes of injured, but not normal spinal cord.

**Conclusion:**

Taken together, these results reveal that after SCI, reactive astrocytes undergo M1 microglia/macrophages-induced necroptosis, partially through TLR/MyD88 signaling, and suggest that inhibiting astrocytic necroptosis may be beneficial for preventing secondary SCI.

**Electronic supplementary material:**

The online version of this article (doi:10.1186/s13024-016-0081-8) contains supplementary material, which is available to authorized users.

## Background

One unique pathological change after primarily spinal cord injury (SCI) is the secondary injury, which is characterized by continuous tissue loss, reactive astrogliosis and chronic inflammation, and usually leads to gradual expansion of the lesion center and formation of a spinal cavity [1, 2]. Elucidating the mechanisms of tissue loss, particularly nerve cell death is important for preventing the expansion of the lesion area. Previous studies have paid attention to the glutamate-induced apoptosis of neurons and oligodendrocytes within and around the lesion center [3–5]. However, how reactive astrocytes, which are the major component of the glial scar, play diverse roles in SCI [6] and are particularly important in supporting neuronal survival [6], are eliminated remains poorly investigated. Understanding the mechanism of astrocytic death post-SCI may yield new insights into understanding the mechanism of secondary SCI and improving functional recovery.

Chronic inflammation plays an essential role in stimulating astrocyte activation and progressive cavitation [7, 8]. After SCI, inflammation is mainly generated by activated microglia/macrophages, which are constituted by their two phenotypically distinct subpopulations, the pro-inflammatory M1, and the anti-inflammatory M2 microglia/macrophages [9]. Compared to the immune reaction in peripheral tissue injury, the polarization of microglia/macrophages post-SCI is M1 predominant and lasts longer [10–12]. Previous study has revealed the apoptosis-inducing effects of M1 microglia/macrophages on neurons and oligodendrocytes [11]. Whether and how the activity of M1 microglia/macrophages affects the survival of reactive astrocytes remains unclear.

In the present study, we analyzed the death of reactive astrocytes in mice and human after spinal contusion, and reported that reactive astrocytes die through necroptosis, a type of programmed necrosis for which the molecular mechanisms have been recently unraveled [13], and is induced by M1 microglia/macrophages, partially via TLR/MyD88 signaling. Our data indicated that blocking the necroptosis of reactive astrocytes might reduce secondary injury and promote functional recovery after SCI.

## Results

### Cavity-surrounding reactive astrocytes undergo necrosis rather than apoptosis or autophagy during the progress of secondary SCI

**Fig. 1:**
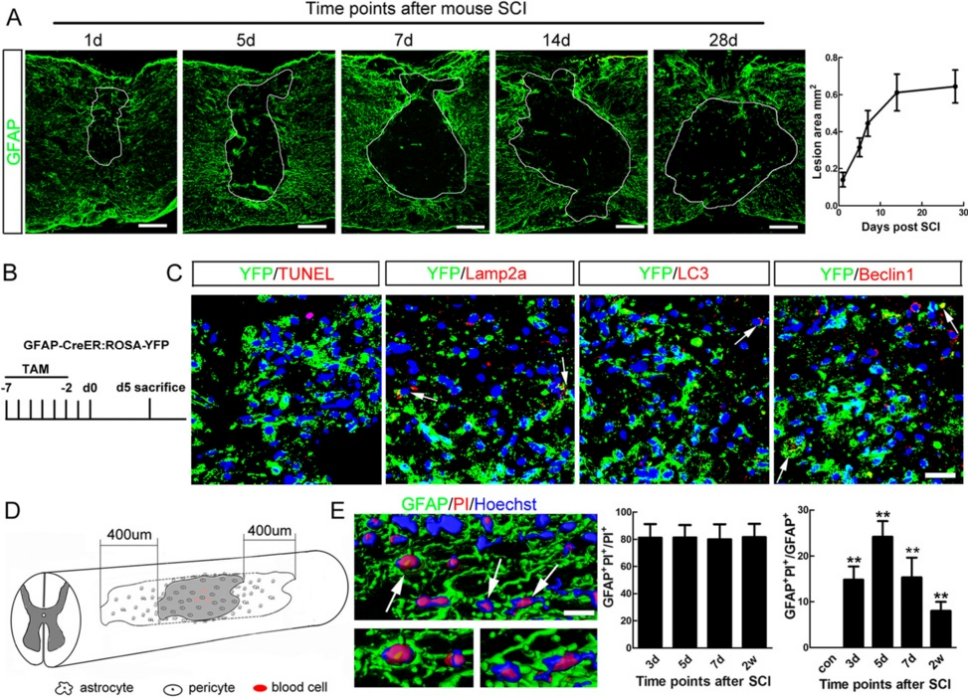
Non-apoptotic or autophagic death of reactive astrocytes after SCI in mice. **a** Immunohistochemistry of GFAP and the size of spinal cavity at different time points after SCI. The spinal cavity enlarges gradually with reactive astrocytes at its border, and reaches plateau at 14 days post-injury in our model. Bar = 200 μm (**b**, **c**) Strategy for astrocytes labeling and Double-staining of YFP with apoptotic marker (TUNEL) and autophagic markers (Lamp2a, LC3 and Beclin-1) at 5 dpi. GFAP-CreER : ROSA-YFP mice were used, and Tamoxifen was injected for 5 successive days before SCI to label astrocytes by YFP. Notice that No double-staining of YFP/TUNEL was found. Only few YFP-positive cells express autophagic markers. Bar = 25 μm. **d** Schematic drawing of morphological quantification area. For quantification of immunostainings, immunoreactivities within two adjacent areas which were 400 μm rostral or caudal to the cavity border in each spinal section were analyzed. **e** 3-D reconstruction of combined PI-labeling and immunohistochemistry of GFAP, and quantification of GFAP- and PI-positive cells. Arrows in C and E point to double positive cells. Bar = 5 μm. **P <0.01. *n* = 3

A modified spinal contusion model which was more experimentally consistent was adopted [14, 15]. Spinal cavity enlarged gradually and reached plateau by 2 weeks post-injury, with reactive astrocytes surrounded at all time points assessed (Fig. 1a). We first tested whether apoptosis or autophagy accounted for the loss of reactive astrocytes during the development of secondary injury. Because immunohistochemistry of GFAP stains primarily processes and seldom cell bodies, we adopted GFAP-CreER:ROSA-YFP mice to visualize the cell bodies of astrocytes. Tamoxifen (2.5 mg) was injected for 5 successive days before SCI to label astrocytes by YFP (Fig. 1b). Double-immunostaining of YFP with TUNEL at 5 days post injury (dpi) showed that very rare could YFP/TUNEL-double positive cells be found, precluding the apoptosis of astrocytes. Double-immunostaining of YFP with autophagic markers showed that in bilateral areas from 400 μm rostral and caudal to border of lesion center defined by YFP/GFAP-immunoreactivity (Fig. 1d), approximately 9.8 % of YFP-positive cells were Lamp2a-positive, 6.3 % were LC3-positive and 8.1 % were Beclin1-positive (Fig. 1c). These data suggest that in mice, during the process of cavitation, reactive astrocytes may be eliminated by ways other than apoptosis and autophagy.

To test whether reactive astrocytes could undergo necrosis, the third major type of cell death, we performed live-animal propidium iodide (PI)-labeling. Besides within lesion center, PI-labeled cells could be found in areas around the epicenter. PI- and GFAP-positive cells in bilateral areas from 400 μm rostral and caudal to border of lesion center defined by GFAP-immunoreactivity were quantified (Fig. 1d). From 3 to 14 dpi, about 80 % of all PI-labeled cells in these regions were GFAP-positive (Fig. 1e). The percent of astrocytes with PI-labeling reached peak at 5 dpi (Fig. 1e). These data indicate that reactive astrocytes around the spinal cavity undergo necrosis during the process of secondary injury.

### Necroptosis of reactive astrocytes after SCI

**Fig. 2:**
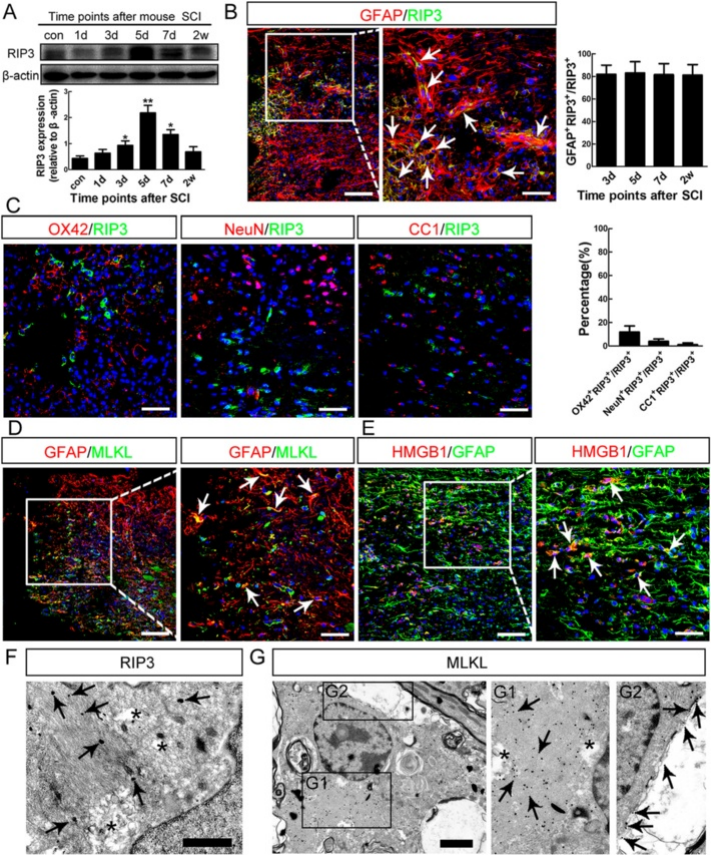
Necroptosis of reactive astrocytes in injured mice spinal cord. **a** Western-blotting of RIP3 after SCI. Notice that the increase of RIP3 from 3 to 7 days post-injury. *P <0.05, **P <0.01. *n* = 3. **b** Double staining and quantification of RIP3 and GFAP at 5 days post-injury. Notice that at all time points, most RIP3-positive cells were GFAP-positive. Arrows points to RIP3/GFAP double-positive cells. Bar = 100 μm. Bar in insert = 50 μm. **c** Double-staining and quantification of RIP3 with OX42, NeuN and CC1. Notice that micoglia, neurons and oligodendrocytes account for a small portion of RIP3-positive cells. Bars = 50 μm. **d**, **e** Double staining of GFAP with MLKL and HMGB1. Many astrocytes around lesion center express MLKL and cytoplasmic HMGB1, shown by *arrows*. Bars = 100 μm. Bars in inset = 50 μm. **f** Immuno-eletron microscopic study of RIP3. Notice the focal cytoplasmic lysis of astrocyte (*asterisks*) and localization of RIP3 immunoreactivity on glial fibrils (*arrows*). Bar = 1 μm. **g** Immuno-eletron microscopic study of MLKL. Notice the fibrils (*arrows* in *G1*) and membrane localization (*arrows* in *G2*) of MLKL. Focal cytoplasmic lysis was shown by asterisks. Bar = 2 μm

Necroptosis is a type of programmed necrosis for which the molecular mechanisms have recently been uncovered [16]. Death signals activate an intracellular signaling which is mediated mainly by protein complexes involving receptor-interacting protein 1 (RIP1), receptor-interacting protein 1 (RIP3) and mixed lineage kinase domain-like protein (MLKL), leading to disruption of cell membranes and lysis of cytoplasmic contents [16, 17]. To test whether necroptosis occurs in the reactive astrocytes after SCI, we first examined the expression of RIP3 in mice by Western-blotting. The expression of RIP3 increased significantly from 3 dpi to 7 dpi (Fig. 2a). Immunohistochemistry detected strong RIP3-immunoreactivity around lesion center, with most expressed by GFAP-positive cells (Fig. 2b). Quantification showed that at all time points examined, approximately 80 % of the RIP3-positive cells were GFAP-positive (Fig. 2b), which was consist with the results of PI-labeling (showed by Fig. 1e). OX42-positive, NeuN-positive, and CC-1-positive cells constituted for the remainder of RIP3-positive cells (Fig. 2c). MLKL, another key molecule in execution of necroptosis [18], was also induced by SCI and expressed in reactive astrocytes (Fig. 2d). In addition, HMGB1, a member of high mobility group box protein that normally binds to chromatin and is released by necrotic cells [19], was detected in the cytoplasm of GFAP-positive cells (Fig. 2e), supporting the occurrence of necrosis in astrocytes.

To confirm the necroptosis of astrocytes after SCI, we performed immune-electron microscopic study in spinal tissue within 5 mm around lesion center at 5 dpi in mice. Astrocytes with focal lysis of cytoplasm and RIP3 immunoreactivity on the cytoplasmic fibrils were frequently found (Fig. 2f). MLKL-immunoreactivity was found both at the cell membrane and within the bundles of cytoplasmic fibrils (Fig. 2g), consistent with its role in penetrating the cell membrane during necroptosis [20]. No apoptotic-like astrocytes were observed. Taken together, these data suggested that reactive astrocytes may undergo RIP3/MLKL-mediated necroptosis after SCI in mice.

### *In vitro* inflammatory stimulation of necroptosis in astrocytes

**Fig. 3:**
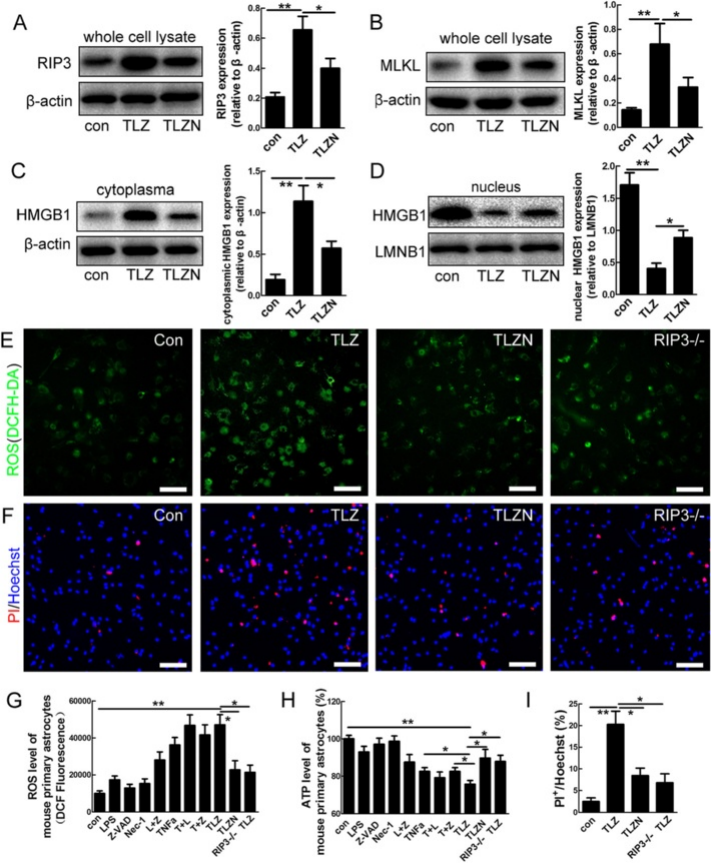
Necroptosis of mouse spinal cord astrocytes *in vitro.* **a**, **b** Western-blotting of RIP3 and MLKL in control, TLZ (TNFα, LPS and Z-VAD), TLZN (TLZ plus Nec-1). Notice that TLZ significantly increased the expression of RIP3 and MLKL. This effect was significantly suppressed by Nec-1. **P <0.01, *P <0.05. *n* = 3. **c**, **d** Western-blotting of cytoplasmic and nuclear HMGB1 in control, TLZ or TLZN-treated astrocytes. Notice that TLZ significantly increased cytoplasmic HMGB1, but decreased nuclear HMGB1. The effects of TLZ on HMGB1 were blocked by Nec-1. **e** ROS staining in control, TLZ treated, TLZN treated and RIP3^−/−^ astrocytes. **f** PI-staining in control, TLZ treated, TLZN treated and RIP3^−/−^ astrocytes. **g**, **h** Quantification of intracellular ROS and ATP levels in astrocytes under various conditions. **i** Percentages of PI-positive cells in TLZ treated, TLZN treated astrocytes and TLZN treated RIP3^−/−^ astrocytes. Bars in (**e**), F = 50 μm. **P <0.01. *P <0.05. *n* = 3

We then examined whether necroptosis could be modeled in reactive astrocytes *in vitro*. Mouse spinal cord astrocytes were cultured and purified as described [21, 22]. Only batches of cells in which the percent of GFAP-positive cells was over 99 % were used for cell death induction (Additional file 1: Figure S1). The cells were challenged with tumor necrosis factor alpha (TNFα) and lipopolysaccharide (LPS) to mimic the inflammatory microenvironment *in vivo,* and z-VAD, a pan-caspase inhibitor was added to inhibit apoptosis as routinely used by researchers when inducing necroptosis [17, 18]. Forty-eight hours treatment of TNFα, LPS and z-VAD (TLZ) significantly increased the expression of RIP3, MLKL, and cytoplasmic HMGB1, while decreased nucleus levels of HMGB1 (Fig. 3a–d). Upon TLZ treatment, the intracellular level of reactive oxygen species (ROS) and the percent of PI-labeled astrocytes were significantly increased, and intracellular level of ATP significantly decreased (Fig. 3e–i). These data indicate that necroptosis of astrocytes can be induced by TLZ *in vitro*. Further, Necrostatin-1 (Nec-1), a well-used necroptosis inhibitor that inhibits phosphorylation of RIP1 [23], significantly compromised the increase of RIP3, MLKL, cytoplasmic high mobility group box 1 (HMGB1), intracellular ROS, extracellular ATP and PI-labeling induced by TLZ treatment (Fig. 3a–i), indicating a rescuing effect of Nec-1 on astrocyte death. Because Nec-1 also inhibits indoleamine-2,3-dioxygenase (IDO) [24], we then used RIP3^−/−^ astrocytes to confirm the occurrence of astrocytic necroptosis. The effects of TLZ on ROS production, ATP level and PI-permeability in wild-type astrocytes were significantly abolished in RIP3^−/−^ astrocytes (Fig. 3e–i). These data demonstrated the necroptosis of mouse astrocytes *in vitro*.

### Inhibiting necroptosis rescues both the death and neurotrophic phenotype of reactive astrocytes after SCI

**Fig. 4:**
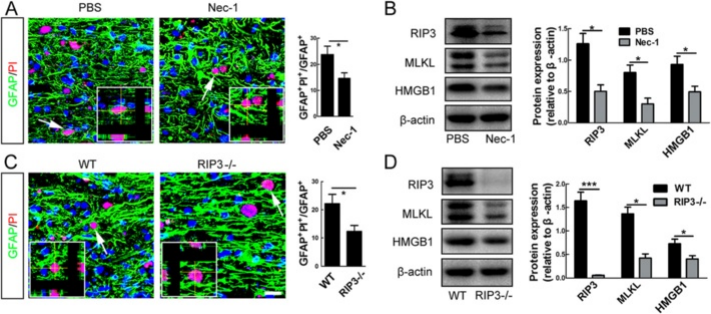
Effects of necroptosis inhibition on astrocyte death after SCI in mice. **a** Combination of PI-labeling with immunohtistochemistry of GFAP in mice treated with PBS or Nec-1 at 5 days post-injury. Nec-1 significantly reduced the percent of PI-positive astrocytes. Bar = 50 μm. *P <0.05. *n* = 3. **b** Western-blotting of RIP3, MLKL and HMGB1 in spinal cord of PBS treated or Nec-1 treated mice at 5 days post-injury. Nec-1 significantly inhibited the expression of RIP3, MLKL and HMGB1, as compared to PBS control. *P <0.05. *n* = 3. **c** Combination of PI-labeling with immunohtistochemistry of GFAP in wild-type (WT) and RIP3^−/−^ mice. RIP3 depletion significantly reduced the percent of PI-positive astrocytes. Bar = 50 μm. *P <0.05. *n* = 3. **d** Western-blotting of RIP3, MLKL and HMGB1 in spinal cord of WT and RIP3^−/−^ mice. RIP3 mutation significantly reduced the SCI-induced expression of RIP3, MLKL and HMGB1. ***P <0.001, *P <0.05. *n* = 3

We next examined the effects of inhibiting necroptosis on the survival of astrocytes after SCI. Five successive days of treatment by Nec-1 significantly decreased the percent of PI-positive astrocytes and the expression of RIP3, MLKL and HMGB1 in areas surrounding lesion center (defined as Fig. 1c), as compared to PBS control (Fig. 4a, b). RIP3^−/−^ mice showed a significant decrease of PI-labeled astrocytes at 5 dpi, as compared to wild type mice (Fig. 4c). The expression of MLKL and HMGB1 was also significantly lower in injured RIP3^−/−^ spinal cord as compared to wild-type control (Fig. 4d). These data indicate that inhibiting necroptosis after SCI could effectively prevent the death of astrocytes.

**Fig. 5:**
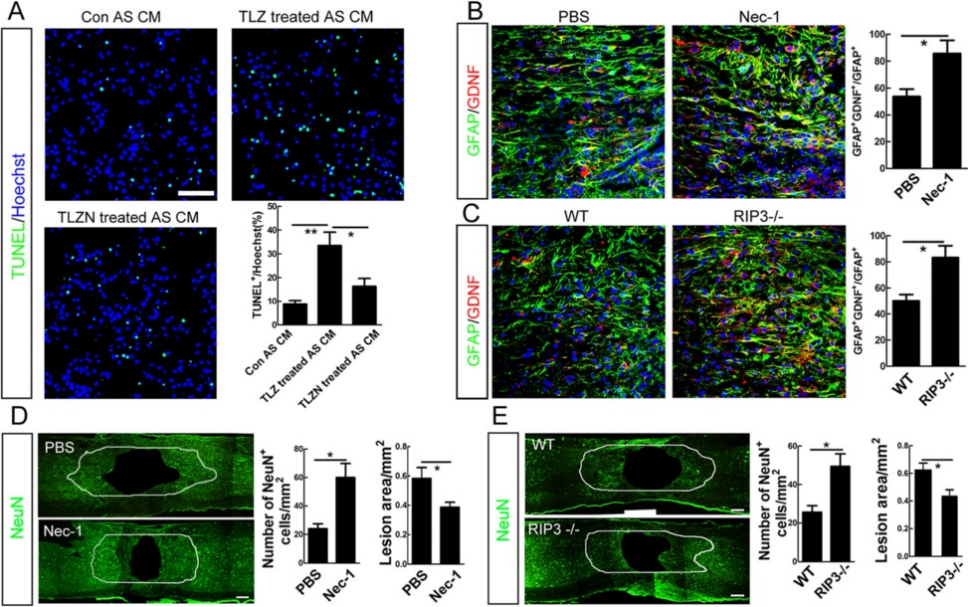
Effects of necroptosis on the neurotrophic function of astrocytes. **a** TUNEL staining of primary spinal cord neurons treated with CM from normal, necroptotic, and necroptosis-inhibited astrocytes. Notice that necroptotic astrocytes were neurotoxic and their detrimental effects on neurons could be alleviated by Nec-1. Bar = 50 μm. **P <0.01, *P <0.05. *n* = 3. **b** Double-staining of GFAP and GDNF in PBS and Nec-1 treated mice. Nec-1 treatment significantly increased the number of reactive astrocytes which express GDNF. Bar = 50 μm. *P <0.05. *n* = 3. **c** Double-staining of GFAP and GDNF in WT and RIP3^−/−^ mice. RIP3 depletion significantly increased the number of reactive astrocytes which express GDNF. Bar = 50 μm. *P <0.05. *n* = 3. **d** Cavity area and the number of neurons in adjacent regions which were 400 μm rostral and caudal to cavity border at 14 days post-injury in mice treated with PBS or Nec-1. Nec-1 significantly improved neuronal survival and reduced cavity area. Bars = 150 μm. *P <0.05. *n* = 6. **e** Cavity area and the number of neurons in adjacent regions which were 400 μm rostral and caudal to cavity border at 14 days post-injury in WT and RIP3^−/−^ mice. RIP3 mutation significantly improved neuronal survival and reduced cavity area. Bars = 150 μm. *P <0.05. *n* = 6

Because astrocytes are well known for their supportive roles in neuronal survival and neurons around the lesion center die after SCI, we then tested whether necroptosis could affect neurotrophic function of reactive astrocytes. Conditioned medium (CM) of normal astrocytes, TLZ treated necroptotic astrocytes, and necroptosis inhibited astrocytes which was treated by TLZ plus Nec-1 (TLZN) were used to treat primary cultured neurons. Twenty-four hours later, more TUNEL-positive neurons were observed in cells treated by CM of necroptotic astrocytes in comparison with those in cells treated by CM of normal astrocytes (Fig. 5a). A significantly less TUNEL-positive neurons were observed in cells treated by CM of TLZN treated astrocytes, as compared to that of TLZ treated astrocytes (Fig. 5a). Further, we examined the effects of necroptosis inhibition on the expression of glial cell line-derived neurotrophic factor (GDNF) by reactive astrocytes after SCI. Both Nec-1 and RIP3 depletion significantly enhanced the expression of GDNF by reactive astrocytes (Fig. 5b, c). We then measured the number of neurons in areas 400 μm rostral and caudal to the spinal cavity at 14 dpi after 7 days treatment by Nec-1 or in RIP3^−/−^ mice, which were thought to undergo apoptosis during secondary injury. The results showed a significant increase of NeuN-positive cells as well as a smaller size of spinal cavity in Nec-1 treated and RIP3^−/−^ mice (Fig. 5d, e). Taken together, these data suggest that inhibiting necroptosis after SCI not only attenuates astrocyte death but also rescues the neurotrophic function of reactive astrocytes, thereby promoting adjacent neuronal survival.

### Induction of necroptosis of reactive astrocytes by M1 microglia/macrophages

**Fig. 6:**
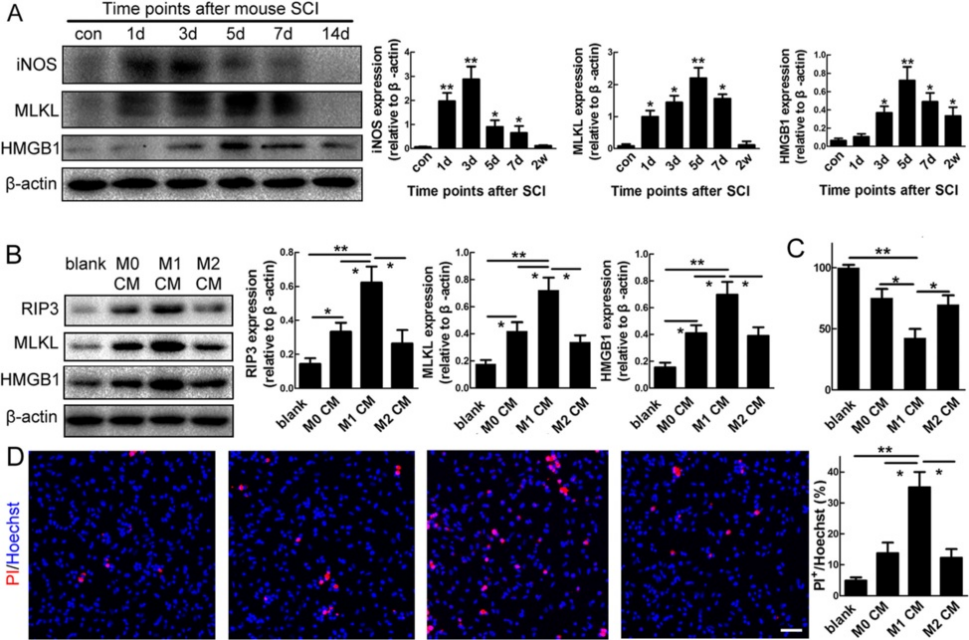
M1 microglia/macrophages induce necroptosis of spinal cord astrocytes *in vitro*. **a** Western-blotting and quantification of iNOS, MLKL and HMGB1 after SCI. Notice the increase of iNOS is followed by the increase of MLKL and HMGB1. *P <0.05, **P <0.01. *n* = 3. **b** Western-blotting and quantification of RIP3, MLKL and HMGB1 in astrocytes treated with conditioned medium (CM) from M0 microglia (M0 CM), M1 microglia (M1 CM), and M2 microglia (M2 CM). Notice that M1 CM has the strongest effects in stimulating the expression of RIP3, MLKL and HMGB1. *P <0.05, **P <0.01. *n* = 3. **c**, **d** Intracellular ATP levels and PI-staining in astrocytes treated by M0 CM, M1 CM, and M2 CM. Dramatic decrease of ATP levels and increase of PI-staining were observed in M1 CM treated astrocytes. *P <0.05, **P <0.01. *n* = 3. *n* = 3. Bar = 50 μm

As inflammatory factors can induce astrocytic necroptosis *in vitro* and M1 microglia/macrophages has been thought to be the major source of toxic inflammatory factors after SCI [12], we hypothesized that M1 microglia/macrophages might induce astrocytic necroptosis after SCI. Western-blotting showed that the expression of inducible nitric oxide synthase (iNOS), a commonly used marker for M1 microglia/macrophages, increased quickly from 1 day and peaked at 3 day post-SCI. The expression of MLKL and HMGB1 increased gradually and peaked at 5 dpi, just following the increase of iNOS (Fig. 6a). To directly investigate the effects of M1 microglia/macrophages on astrocytes, we polarized primarily cultured microglia/macrophages toward either M1 or M2 phenotype, and stimulated astrocytes with conditioned medium collected from M0 (normal cultured microglia/macrophages), M1, and M2 microglia/macrophages. The polarization of microglia or macrophages was confirmed by the expression of iNOS and arginase 1, a typical marker for M2 microglia/macrophages (Additional file 2: Figure S2). All three CMs increased the expression of RIP3, MLKL and HMGB1 in cultured astrocytes. Of note, CM of M1 microglia/macrophages (M1 CM) showed the strongest induction of these necroptosis markers (Fig. 6b, Additional file 3: Figure S3). In addition, intracellular ATP was significantly decreased, and the number of PI-labeled cells increased in astrocytes treated by M1 CM (Fig. 6c, d).

**Fig. 7:**
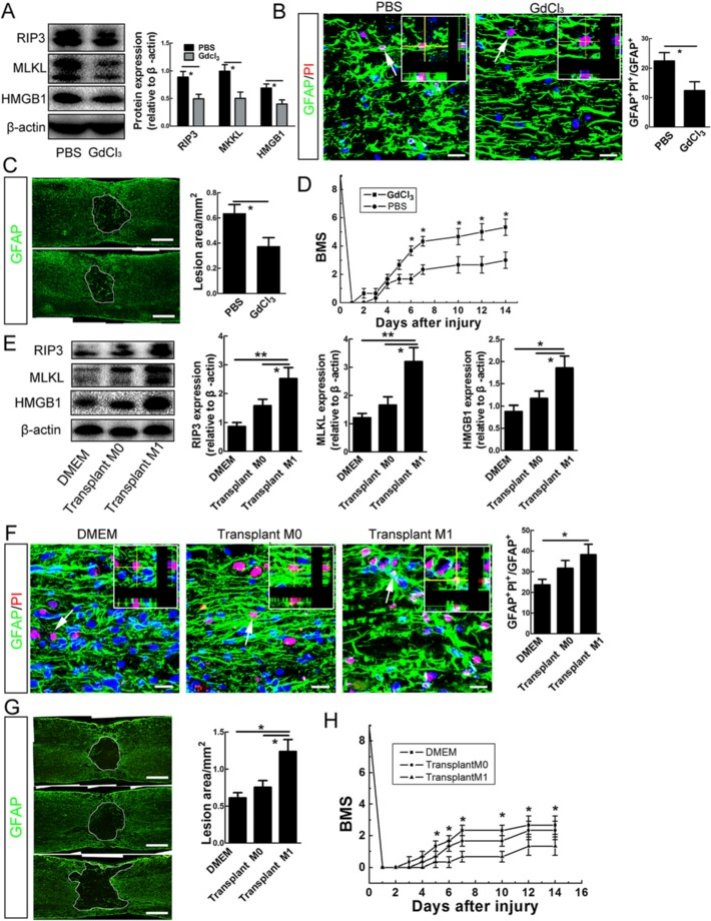
Effects of depleting M1 microglia/macrophages or transplantation of M1 macrophages on astrocytic death and functional recovery. **a** Western-blotting ofRIP3, MLKL and HMGB1 at 5 days post-injury in GdCl_3_- or PBS- treated mice. GdCl_3_ treatment significantly blocked the induction of RIP3, MLKL and HMGB1 by SCI. *P <0.05. *n* = 3. **b** Double-staining of GFAP and PI at 5 days post-injury in GdCl_3_ or PBS treated mice. GdCl_3_ treatment significantly reduced the percent of PI-labeled astrocytes. Bar = 50 μm. *P <0.05. *n* = 3. **c**, **d** Quantification of cavity area at 14 days post-injury and evaluation of locomotion recovery in GdCl_3_ and PBS treated mice. GdCl_3_ treatment significantly reduced cavity size and enhanced functional recovery. BMS stands for for Basso mouse scale. Bars = 200 μm. *P <0.05. *n* = 3. **e**, **f** Western-blotting of RIP3, MLKL and HMGB1, and double-staining of GFAP and PI in injured spinal cord at 5 days after transplantation of M0 macrophages or M1 macrophages. M1 macrophages significantly increased the expression of RIP3, MLKL and HMGB1, and the number of PI-labeled astrocytes, as compared to DMEM control. Bar = 50 μm. **P <0.01, *P <0.05. *n* = 3. **g**, **h** Quantification of cavity area and locomotion recovery after transplantation of DMEM, M0 macrophages or M1 macrophages. Transplantation of M1 macrophages significantly expanded the area of spinal cavity and impeded function recovery. BMS stands for for Basso mouse scale. *P <0.05. *n* = 3. Bars = 200 μm

To investigate the death-inducing effects of M1 microglia/macrophages on astrocytes *in vivo*, we depleted M1 microglia by administration of gadolinium chloride (GdCl_3_) in the lesion site as described [25], which induces apoptosis of inflammatory macrophages via competitive inhibition of Ca^2+^ mobilization and damage to plasma membranes [26, 27]. Depletion of M1 microglia/macrophages was confirmed by reduced expression of iNOS (Additional file 4: Figure S4). In comparison with PBS control, GdCl_3_ treatment significantly reduced the expression of RIP3, MLKL and HMGB1, and decreased the percent of PI-labeled astrocytes within bilateral regions 400 μm rostral and caudal to the epicenter (Fig. 7a, b). Consistently, smaller spinal cavity at 14 dpi and better locomotion recovery from 6 dpi were observed in GdCl_3_ treated mice (Fig. 7c, d).

To further confirm the effects of M1 microglia/macrophages on astrocyte death and spinal cavity, we prepared primary cultured macrophages from bone marrow, and transplanted M0 and M1-polarized macrophages into the injured spinal cord. Transplantation of M1 macrophages resulted in a dramatic increase of RIP3, MLKL and HMGB1 expression and the number of PI-labeled astrocytes, while transplantation of M0 macrophages only showed minor effects, as compared to Dulbecco’s Modified Eagle’s Medium (DMEM) control (Fig. 7e, f). Likewise, the spinal cavity was significantly larger at 14 dpi, and the locomotion recovery worse from 5 dpi in M1 macrophage-transplanted mice (Fig. 7g, h). Taken together, these data indicate that M1 micoglia/macrophages play an inductive role in the necroptosis of astrocytes after SCI.

### Involvement of astrocytic TLR/MyD88 signaling in M1 microglia/macrophage-induced necroptosis

**Fig. 8:**
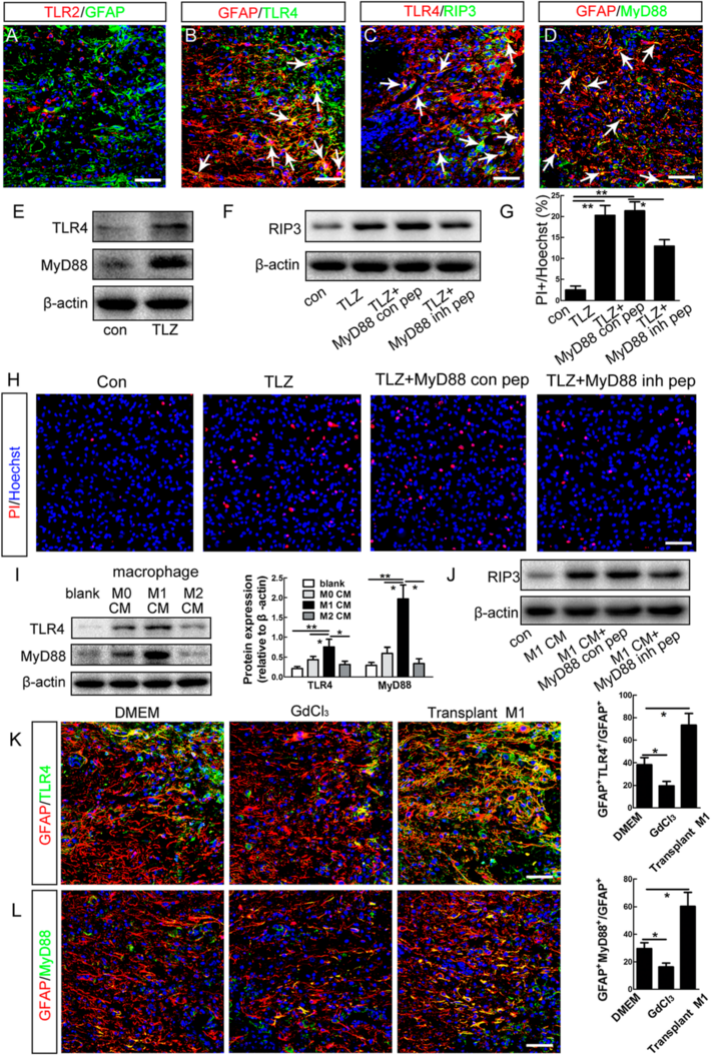
Involvement of TLR4/MyD88 signaling in M1 microglia/macrophages-induced necroptosis of astrocytes. **a**, **b** Double-staining of GFAP with TLR2 or TLR4. Notice that many GFAP-positive cells around the lesion center express TLR4 (*arrows*). Bars = 50 μm. **c**, **d** Double-staining of RIP3/TLR4 and MyD88/GFAP. Notice that there are many RIP3/TLR4 and MyD88/GFAP double-positive cells (showed by *arrows*) in the injured mouse spinal cord. Bars = 50 μm. **e** Western-blotting of TLR4 and MyD88 in cultured astrocytes after treatment with TLZ or vehicle control. TLZ treatment dramatically induced the expression of TLR4 and MyD88. **f**–**h** Western-blotting of RIP3 and PI-staining in astrocytes treated with vehicle control, TLZ, TLZ plus control peptide (MyD88 con pep), and TLZ plus a MyD88 inhibitory peptide (MyD88 inh pep). Notice that MyD88 inhibitory peptide significantly suppressed the effects of TLZ on the expression of RIP3 and MLKL and PI-labeling. **P <0.01, *P <0.05. *n* = 3. Bar = 50 μm. **i** Western-blotting of TLR4, MyD88 in normally cultured astrocytes, and astrocytes treated with M0 CM, M1 CM, and M2 CM from microglia/macrophages. Notice that M1 CM significantly increased the expression of TLR4 and MyD88. **P <0.01, *P <0.05. *n* = 3. **j** Western-blotting of RIP3 in astrocytes treated with DMEM control, M0 CM, M1 CM, and M2 CM. **k**, **l** Double-staining of GFAP with TLR4 and MyD88 at 5 dpi in DMEM injected, GdCl_3_ treated or M1 macrophages transplanted mice. Notice that GdCl_3_ treatment significantly decreased, while transplantation of M1 macrophages significantly increased the percent of astrocytes expressing TLR4 or MyD88 in adjacent regions which were 400 μm rostral and caudal to cavity border. *P <0.05. Bars = 50 μm. *n* = 3

As showed above, LPS was required for the *in vitro* induction of astrocytic necroptosis. We thus speculated that toll-like receptors (TLR) and their downstream molecules might be involved in the M1 microglia/macrophages-induced astrocytic necroptosis. Among TLRs members, TLR2 and TLR4 respond to same type of stimulus and have been thought to be involved in astrocyte activation [28, 29]. Double-staining of GFAP with TLR2 or TLR4 showed that only a minor portion of reactive astrocytes surrounding spinal cavity express TLR2, while most reactive astrocytes express TLR4 (Fig. 8a, b). We then focused on the expression of TLR4 and the intracellular adaptor molecule MyD88. The immunoreactivity of TLR4 overlapped very well with that of RIP3 in the injured mouse spinal cord (Fig. 8c). MyD88 was also highly expressed in reactive astrocytes (Fig. 8d), suggesting that TLR4/MyD88 signaling was activated in necroptotic astrocytes after SCI. *In vitro*, TLZ treatment stimulated a dramatic increase of TLR4 and MyD88, as well as a moderate increase of TLR2, in astrocytes (Fig. 8e, Additional file 5: Figure S5). A TAT-tagged MyD88 inhibitory peptide which could block the homodimerization of MyD88 was added into culture medium of astrocytes to antagonize the function of MyD88 [30]. The MyD88 inhibitory peptide significantly suppressed the increase of RIP3 and PI-labeling in astrocytes induced by TLZ treatment (Fig. 8f–h), suggesting that TLR/MyD88 may be required for the activation of necroptotic signaling in astrocytes.

We then examined the effects of M1 microglia/macrophages on the expression of TLR4 and MyD88 in astrocytes. Conditioned medium from M1 macrophages significantly increased the expression TLR4 and MyD88 (Fig. 8i). The MyD88 inhibitory peptide effectively blocked the induction of RIP3 by M1 macrophages (Fig. 8j). *In vivo*, GdCl_3_ treatment significantly decreased the percent of astrocytes expressing TLR4 or MyD88 by approximately 39 and 47.4 %, respectively (Fig. 8k, l). In contrast, transplantation of M1 macrophages significantly increased the number of astrocytes expressing TLR4 and MyD88, compared to the DMEM control (Fig. 8k, l). These data indicate that in mice, TLR/MyD88 signaling may be involved in the M1 microglia/macrophage-induced necroptosis of astrocytes.

### Expression of necroptotic markers and TLR4/MyD88 in astrocytes of injured human spinal cord

**Fig. 9:**
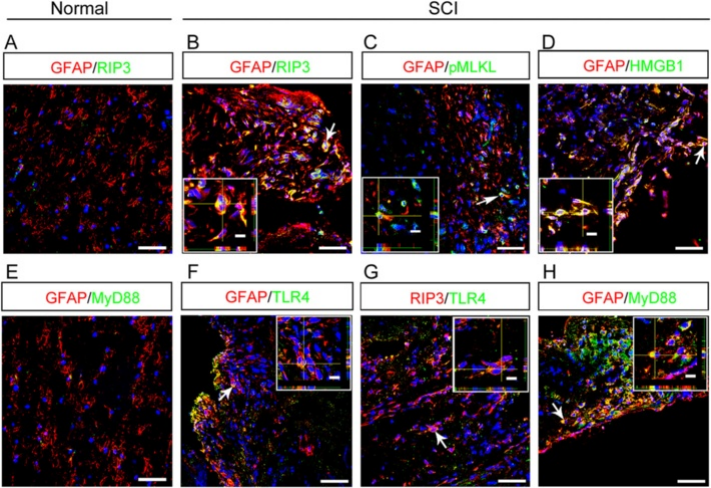
Expression of necroptotic markers and TLR/MyD88 by astrocytes in injured human spinal cord. **a** Double-staining of GFAP with RIP3 in uninjured human spinal cord. Notice the very weak expression of RIP3. Bar = 50 μm. **b**–**d** Representative images of double-staining of GFAP with RIP3, pMLKL and HMGB1 in human spinal cord at 5 days post-injury. Notice the co-localization of RIP3, pMLKL and HMGB1 with GFAP. Bars = 50 μm. **e** Double-staining of GFAP with MyD88 in uninjured human spinal cord. Notice the very weak expression of MyD88. Bar = 50 μm. **f**–**h** Representative images of double-staining of TLR4/RIP3, TLR4/GFAP and GFAP/MyD88 in human spinal cord at 5 and 15 days post-injury. Notice the co-localization of TLR4 with RIP3 and GFAP, and MyD88 with GFAP. Bars = 50 μm

The above experiments in mice indicated that after SCI, reactive astrocytes died by necroptosis, which was induced by M1 microglia/macrophages, partially through TLR/MyD88 mediated signaling. We next tested whether similar pathological changes could occur in human after SCI. Immunohistochemistry showed a very weak expression of RIP3 and MyD88, mainly by astrocytes in normal human spinal cord (Fig. 9a, e). No expression of MLKL, HMGB1 and TLR4 was detected in normal human spinal cord (data not shown). However, after SCI, strong RIP3-, phosphorylated-MLKL- (pMLKL) and HMGB1-immunoreactivities were detected in GFAP-positive cells around the lesion center in one patient at 5 dpi (Fig. 9b–d). Interestingly, TLR4- and MyD88-immunoreactivities were also detected in RIP3-positive or GFAP-positive cells (Fig. 9f–h). Similar expression patterns of RIP3, pMLKL, HMGB1, TLR4 and MyD88 were observed in another patient at 15 dpi (data not shown). These results suggested that in human, reactive astrocytes may also undergo necroptosis after SCI.

## Discussion

Necrosis has been traditionally thought to account for the acute cell loss post-SCI and be uncontrollable [31]. Recent progress in the field of cell death has identified a novel type of programmed necrosis, necroptosis [23], and unveiled the underlying molecular mechanism, which is mediated by an intracellular RIP1/3/MLKL signaling cascade [32, 33], thereby offering an opportunity for re-examining necrosis after SCI. Previous studies have reported protective effects of Nec-1 on SCI in rats without knowing the cell types that Nec-1 targets [34, 35]. Our *in vivo* PI-labeling showed that astrocyte was the major type of cells that undergo necrosis after SCI. The ultrastructural localization of RIP3 and MLKL on the cytoplasmic glial fibrils confirmed the astrocytic necroptosis in SCI. Interestingly, PI-positive astrocytes persisted for 2 weeks in injured spinal cord, indicating that chronic necrosis may be an important contributor of cavity formation post-SCI. We recently reported that microglia/macrophages undergo necroptosis after SCI [36], which was consistent with our observation that RIP3 was primarily expressed by reactive astrocytes, and secondarily expressed by microglia/macrophages (Fig. 2b, c). Considering the fact that inflammation plays critical roles in the cavity formation after SCI, the beneficial effects of necroptosis inhibition on SCI may be results from the protection of astrocytes, microglia/macrophages, as well as other cells.

The innate immune reaction produced by microglia/macrophages has been demonstrated to contribute to the cavity formation and enlargement after SCI [7]. The destructive effects of activated microglia/macrophages were largely attributed to their M1 sub-group, which was activated quickly after SCI and expressed high levels of pro-inflammatory cytokines, including the well-studied necroptosis inducing factor TNFα [23, 37]. It is therefore reasonable to speculate a link between M1 micorglia/macrophages and astrocyte death after SCI, which has been poorly investigated. Our data showed that *in vitro,* conditioned medium of M1 microglia/macrophages could induce necroptosis of astrocytes. *In vivo*, depletion of M1 microglia/macrophages by GdCl_3_ or transplantation of M1 macrophages can reduce or enhance necroptosis of astrocytes respectively. These results indicated a critical role of M1 microglia/macrophages in inducing the necroptosis of astrocytes after SCI. Considering that GdCl_3_ also affects neutrophils [38], which are abundant in the injury epicenter after SCI, and that iNOS can also be expressed by neutrophils [39]. The beneficial effects of GdCl_3_ treatment may also be contributed by the inhibition of neutrophils.

Although the identities of death factors released by M1 microglia/macrophages remain unclear, our data showed that TLR4 and MyD88 were up-regulated in necroptotic astrocytes after SCI and M1 CM could increase the expression of TLR2, TLR4 and MyD88 in astrocytes*.* Previous researches have reported that TLR4 is involved in the necroptosis of macrophages *in vitro* and in the activation of astrocytes after SCI [40, 41]. These results indicated that M1 microglia/macrophages may induce the necroptosis of astrocytes by activating TLR4/MyD88 signaling. In consistent, inhibiting MyD88 could partially block the necroptosis-inducing effect of M1 microglia/macrophages *in vitro*. In addition, the expression of TLR4/MyD88 in human necroptotic astrocytes indicated a common response of this signaling pathway after SCI. Considering that MyD88 mediates the downstream signals of multiple TLRs, and TLZ stimulates both the expression of TLR2 and TLR4 *in vitro*, the involvement of other TLRs in the M1 microglia/macrophages-induced astrocytic death is not excluded. It has been demonstrated that necroptotic cells release factors that modulate inflammation [42], whether necroptotic astrocytes could regulate the chronic inflammation after SCI is of interest to be further investigated.

As the major component of glial scar, reactive astrocytes exhibit heterogeneous properties and exert multi-faceted functions in SCI, such as providing nutritive and metabolic support for neurons, inhibiting axonal growth and modulating inflammation [43, 44]. Our data showed that necroptotic astrocytes were less supportive for neuronal survival, and inhibiting astrocytic necroptosis could rescue the neurotrophic function of reactive astrocytes, thereby reducing cavity area and promoting the survival of neurons surrounding lesion center which otherwise underwent apoptosis during the secondary injury [45, 46]. It is still unknown how the properties of reactive astrocytes change when necroptotic signaling is activated. Nevertheless, our data, for the first time, have revealed a novel mechanism for the astrocytic death after SCI, implying that astrocytic necroptosis may be manipulated for preventing secondary SCI in the future.

## Conclusions

After SCI, cavity-surrounding reactive astrocytes undergo RIP3/MLKL-mediated necroptosis, rather than apoptosis and autophagy. The necroptosis of astrocytes is induced by M1 microglia/macrophages, partially through TLR/MyD88 signaling. Reactive astrocytes in injured human spinal cord die through similar mechanism. Our data suggested that inhibiting astrocytic necroptosis may be beneficial for preventing secondary SCI.

## Methods

### Animals and human samples

RIP3^−/−^ mice were generated as described [47]. GFAP-CreER and ROSA-YFP mice were bought from Jackson laboratory. Wild-type mice were bought from the animal center of the Fourth Military Medical University. All protocols of animal experiments were approved by the Animal Care and Use Committee of the Fourth Military Medical University.

Snap-frozen normal human spinal cord tissues were obtained from human brain bank of school of medicine at Zhejiang University. Biopsy of injured spinal cord tissues were performed with informed consent obtained from each patient prior to surgery and experiments involving human spinal tissues were approved by the Institutional Review Board of Tangdu Hospital, Fourth Military Medical University.

### Reagents

Necrostatin-1 was bought from Tocris. Propidium iodide, DCFH-DA, GdCl_3_ and LPS were bought from Sigma. ATP detecting and TUNEL staining kits were from Promega. LPS, z-VAD and human TNFα were from R & D system. IFN-γ and IL-4 were from Peprotech. The source and dilution of antibodies were included in Table 1.

**Table 1:** Information for primary antibodies used

Antibodies	Hosts	Dilutions	Sources
Anti-Arginase1	Goat	IHC 1:50, WB 1:200	Santa Cruz
Anti-Beclin1	Mouse	IHC 1:200, WB 1:500	Cell Signaling
Anti-β-actin	Mouse	WB 1:50000	Sigma
Anti-CC1	Mouse	IHC1:500	Millipore
Anti-GFAP	Mouse	IHC1:500	Millipore
	Rabbit	IHC1:1000	Dako
Anti-HMGB1	Rabbit	IHC 1:100, WB 1:500	Proteintech
Anti-iNOS	Mouse	IHC 1:200, WB 1:500	BD
Anti-Lamp2a	Rabbit	IHC1:200	Abcam
Anti-LC3	Mouse	IHC1:500	Cell Signaling
Anti-MLKL	Rat	IHC 1:200, WB 1:500	Millipore
anti-pMLKL	Rabbit	IHC1:300	Abcam
Anti-MyD88	Rabbit	IHC 1:50, WB 1:200	Abcam
Anti-NeuN	Mouse	IHC1:500	Millipore
Anti-OX42	Mouse	IHC1:200	Abcam
Anti-RIP3	Rabbit	IHC 1:200, WB 1:500	Enzo
	Rabbit	WB 1:500	Abcam
Anti-TLR2	Rabbit	IHC1:100, WB 1:200	EPITOMICS
Anti-TLR4	Mouse	IHC 1:200, WB 1:500	Abcam
Anti-YFP	Goat	IHC1:600	Rockland

### Spinal cord contusion and *in vivo* treatments

C57 mice of 25–30 g were anesthetized with 1 % sodium pentobarbital. For improving the consistency of the experimental data, W. Tazlaff developed a manual graded forceps lateral crush SCI model [48]. Because it is difficult for manual performance to ensure vertical orientation of the forceps and equal degree of compression of the two sides of the cord, we designed a mechanical version by mounting a pair of forceps on a stereotaxic device, and thereby its two blades could be closed simultaneously from both sides [14]. Briefly, a 15–30 mm dorsal midline incision was made by bilateral laminectomy. Spinal cord injury was made at T8 vertebra (corresponding to T9 segment of the spinal cord) by lateral crushing, setting the gap between the blades of the forceps at 0.2 mm for 15 s.

#### In vivo Propidium iodide (PI) staining

PI (10 mg/ml) was diluted in 0.9 % NaCl. Twenty mg/kg of PI in a total volume of not more than 100 μl was administered (i.p.) to mice 1 h before sacrifice as described [49, 50].

#### Nec-1 administration

Nec-1 (7.8 mg/kg) was administrated intravenously (i.v.) twice a day for 5 or 7 days (5 days for examining the expression of necroptotic markers, and 7 days for evaluating cavity size and locomotion recovery).

#### Genetic astrocyte labeling

Tamoxifen (2.5 mg) in corn oil was administered from 7 days before SCI for five successive days (Fig. 1b).

#### M1 microglia/macrophages depletion

One day after SCI, GdCl_3_ (270 μM) was injected into the lesion area as described [25]. Three injections were made, one in the lesion center, two at 2 mm rostral or caudal to the epicenter, respectively. The volume of each injection is 1.5 μl. The efficiency of depletion was confirmed 3 days after injection by western-blotting of iNOS.

#### M1 macrophages transplantation

Immediately after SCI, a total of 2 × 10^6^ M1, M0 microglia in 2 μl, or same volume of Dulbecco’s modified Eagle’s medium (DMEM) was injected into the lesion center.

#### Behavior evaluation

The Basso Mouse Scale (BMS) was used to rate locomotor function and recovery by two investigators blinded to the experimental design.

### Cell culture and *in vitro* treatments

#### Astrocyte culture, purification and treatments

Mice at postnatal day 2–4 were freeze anesthetized, and skin sterilized with 75 % alcohol. The brain was removed under a stereomicroscope. The cortex was dissected and meninges peeled off. The tissue was digested in 0.125 % trypsin for 10 min at 37 °C and the digestion was stopped by adding 10 % fetal bovine serum (FBS) in DMEM. After centrifuging at 800 rpm for 5 min, the cell suspension was moved to 75 cm flasks pre-coated with poly-D-lysine and cultured in DMEM containing 10 % FBS at 37 °C with 5 % CO_2_. Culture medium was half changed every other day.

Astrocytes were purified as described with modifications [21, 22]. When cells reached confluence, the culture was purified by shaking at 260 rpm overnight, and the suspended microglia and oligodendrocytes were discarded. The cells were then cultured with 8 μM arabinoside C (Ara C) for 3 days, followed by a 1 h l-leucine methyl ester (LME, 60 μM) treatment. This cell shaking, passaging, Ara C and LME treatment were repeated once. Immunocytochemistry of GFAP, Sox10 and CD11b was performed to evaluate the purity of astrocytes. Only batches of cells with GFAP-positive cells over 99 % were used for cell death induction.

For necroptosis induction, TNFα (100 ng/ml), LPS (4 μg/ml) and z-VAD (20 μM) were used according to literatures [23, 51, 52] and our preliminary experiments, and added to the culture medium for 48 h. For collecting CM from necroptotic astrocytes, TLZ was washed off before fresh serum-free medium was added. And then CM was collected 24 h later. For necroptosis inhibition, Necrostatin-1 (20 μM) was added together with TNFα, LPS and z-VAD (TLZ) stimulation. For interfering with TLR4/MyD88 signaling, MyD88 inhibitory peptide (100 μM, Novas, Cat. NBP2-29328) was added into medium 8 h before TLZ treatment.

#### Micoglia culture and treatments

When primarily cultured astrocytes reach confluency, cells were shaken at 260 rpm for 30 min. The cells in the suspension were collected and re-plated. The purity of microglia was confirmed by immunostaining of CD11b. For inducing M1 microglia, cells were treated with LPS (100 ng/ml) plus IFN-γ (20 ng/ml) for 24 h. For M2 polarization, cells were treated with IL-4 (20 ng/ml) for 24 h. Then stimulators were removed and medium refreshed for 24 h before collecting conditioned medium. The normal cultured microglia (M0) received no treatment except regular medium refreshment.

#### Spinal cord neuron culture and treatments

Spinal cords were dissected from E12-13 mice embryos. The dorsal root ganglions and meninges were carefully peeled off. The tissue was digested in 0.125 % trypsin for 10 min at 37 °C and the digestion was stopped by adding 10 % fetal bovine serum (FBS) in Neurobasal. After centrifuging at 700 rpm for 6 min, the cell suspension was moved to 25 cm culture dishes pre-coated with poly-D-lysine and cultured in Neurobasal containing 1 % N2 supplement and 8 μM Ara C. Forty-eight hours later, Ara C was washed off, and neurons cultured with Neurobasal containing 1 % N2. Culture medium was half changed every other day.

Seven days after culture, neurons were cultured with conditioned medium from TLZ treated astrocytes or control astrocytes for 24 h. TUNEL staining was then performed for assessing cell death.

#### Bone marrow macrophage culture and treatment

Bone marrow cells were collected from the femurs and tibias of mice by trituration using 26-gauge needles. Red blood cells were lysed by lysis buffer containing 0.15 M NH_4_Cl, 10 nM KHCO_3_, and 0.1 mM EDTA (pH7.4). After washing with RPMI 1640, the cells were cultured in RPMI 1640 supplemented with 1 % penicillin/streptomycin, 1 % 4-(2-hydroxyethyl) piperazine-1-ethanesulfonic acid, 0.1 % β-mercaptoethanol, 10 % FBS, and 20 % sL929-conditioned medium containing macrophage colony-stimulating factor (M-CSF). After 7–10 days culture, nonadherent cells were removed. Adherent cells were treated with LPS (100 ng/m) and IFN-γ (20 ng/ml) for M1 polarization, or with IL-4 (20 ng/ml) for M2 polarization. Twenty-four hours later, the stimulators were removed and medium refreshed before collecting conditioned medium. The normal cultured macrophages were considered as M0 macrophages.

### Immunohistochemistry

Animals were sacrificed and perfused intracardially with 4 % cold paraformaldehyde phosphate buffer (pH 7.4). Following perfusion, a 2 cm spinal cord segment with the lesion site at its center was removed and cryoprotected by 25 % sucrose**.** For each mice, serial sections (20 μm in thickness for each section) were cut and all the sections were collected onto eight slides. Among these eight slides, only 2–3 slides contain one section which was cut through central canal. For immunostaining, the sections were blocked by 0.01 M phosphate buffered saline (PBS) containing 0.3 % Triton X-100 and 3 % bovine serum albumin (BSA) for 1 h. Primary antibodies (as described in Table 1) were incubated at room temperature overnight. After washing with PBS, sections were incubated with their corresponding secondary antibodies conjugated with Alexa Fluor 594 (donkey anti-rabbit or anti-rat IgG, 1:800, Molecular probes), Alexa Fluor 488 (donkey anti-mouse, 1:500, Molecular probes) or Alexa Fluor 680 (donkey anti-rabbit IgG, 1:1000, Molecular probes) for 4 h at room temperature protected from light. The nuclei were counterstained by Hoechst33342 (1:5000, Sigma). All immunostained sections were photographed under a confocal microscope (FV1000, Olympus) with same setting. 3-D reconstruction was made using IMARIS software.

Quantification of the immunostaining in injured spinal cord was performed as described [14, 53, 54]. One of the slides which contain the section cut through central canal was randomly chose for immunostaining, and all the sections on this slide were subjected to quantification. The lesion area was defined by the inner lining of GFAP stained astrocytes. After outlining the injury epicenter, the borderline was shifted 400 μm rostrally and caudally respectively (Fig. 1c). The resulting areas from shifting were calculated by converting the pixels into millimeters by using Image J. All the immunopositive cells within the defined area were counted by using ImagePro Plus Version 5.0. The slide selection and cell counting were performed by an investigator who was blind to experimental design.

#### TUNEL staining

For TUNEL/GFAP double-staining, TUNEL staining was performed first according to the manual of DeadEND™ TUNEL system (Promega), and then followed by immunostaining of GFAP.

### Immuno-electron microscopy

The immune-electron microscopic study was performed as described [55]. Briefly, at 5d after SCI, the mice were perfusion fixed with a mixture of 4 % paraformaldehyde, 0.05 % glutaraldehyde, and 15 % saturated picric acid for 30 min. Then injured spinal cords were removed and postfixed in the same fixative without glutaraldehyde for 3 h. Tissue sections of 50 μm were prepared with a vibratome and cryoprotected by 30 % sucrose. After one freeze-thaw treatment, the sections were blocked by 5 % bovine serum albumin and 5 % normal goat serum, incubated with anti-RIP3 or MLKL antibodies, and then with goat anti-rabbit or anti-rat IgG conjugated to 1.4 nm gold particles (1:100, Nanoprobes) at room temperature overnight sequentially. After rinsing, the sections were postfixed with 2 % glutaraldehyde for 45 min. Silver enhancement was performed in the dark with an HQ Silver Kit (Nanoprobes). The sections were further fixed with 0.5 % osmium tetroxide, dehydrated with graded ethanol, replaced with propylene oxide, and flat-embedded in Epon 812. The RIP3- and MLKL-immunoreactive areas surrounding lesion center were selected, trimmed under a stereomicroscope and mounted onto blank resin stubs for ultrathin sectioning. Ultrathin sections (70–80 nm) were prepared on an LKB Nova Ultratome (Bromma). After being counterstained with uranyl acetate and lead citrate, the sections were examined under a JEM-1230 electron microscope (JEM, Tokyo).

### Western blot analysis

The spinal cord segments with lesion at their center were dissected out. Each sample was homogenized in RIPA buffer for about 20 min and incubated for another 40 min on ice, then centrifuged at 12 000 g at 4 °C. Supernatant was boiled before sodium dodecyl sulfate polyacrylamide gel electrophoresis. The proteins were electrotransferred to polyvinylidene difluoride membrane and reacted with primary antibodies ( as described in Table 1) overnight at 4 °C, then with corresponding secondary anti-mouse, anti-rabbit, or anti-rat IgG-peroxidase (1:5000) at room temperature for 50 min. The bands were visualized by an ECL kit (Millipore).

### *In vitro* PI-staining, ROS and ATP measurement

#### Live cell PI labeling

PI (5 μM) and Hoechst 33342 (5 μg/ml) were added into the culture medium and incubated for 30 min at 37 °C. Cells were then washed three times with 0.01 M PBS and fixed with 4 % (w/v) paraformaldehyde in PB for 10 min at room temperature and then imaged under an inverted fluorescence microscope (IX71, Olympus) equipped with an Olympus DP72 digital camera.

#### ROS measurement

Oxidation-sensitive fluorescent probe DCFH-DA was adopted to evaluate ROS levels. Astrocytes were cultured in 96-well plates. DCFH-DA (10 μM) was added and incubated for 20 min at 37 °C. After washing with PBS, the ROS levels were determined by a multimode microplate reader (TECAN, infinite M200), and images were photographed under an inverted fluorescence microscope (IX71, Olympus).

#### ATP measurement

Intracellular levels of ATP were measured using the Cell Titer-Glo luminescent cell viability assay kit (Promega) according to the manufacturer’s instructions. Luminescence was measured by multimode microplate reader (TECAN, infinite M200).

### Statistical analysis

For each of triplicate *in vitro* experiments, pictures were taken from eight random fields. All cells in the images were analyzed. Image Tool (University of Texas Health Sciences Center at San Antonio) was used for quantification.

The data were presented as means ± S.E., and analyzed by one-way ANOVA, followed by Dunnett post hoc, except for BMS scoring which was further analyzed by Bonferroni post hoc using SPSSl6.0 (Chicago, IL, USA) as recommended [56]. *P* values less than 0.05 were considered as statistical significant.

## Additional files

Additional file 1: Figure S1. Double-staining of GFAP with Sox10 and CD11b after astrocyte purification. Over 99 % of cells were GFAP-positive. Very rare could Sox10- and CD11b-positive cells be detected. Bar = 15 μm. (TIF 2385 kb) Additional file 2: Figure S2. Western-blotting of iNOS and Arginase 1 in M1 and M2 polarized micoglia and macrophages. (TIF 1374 kb) Additional file 3: Figure S3. Western-blotting and quantification of RIP3, MLKL and HMGB1 in astrocytes treated by conditioned medium from M0 microglia (M0 CM), M1 microglia (M1 CM), and M2 microglia (M2 CM). Notice that M1 CM has the strongest effects in stimulating the expression of RIP3, MLKL and HMGB1. **P* <0.05, ***P* <0.01. *n* = 3. (TIF 3027 kb) Additional file 4: Figure S4. Western-blotting of iNOS at 5 dpi in GdCl_3_ or PBS treated mice. The expression of iNOS was reduced in GdCl_3_ treated mice. **P* <0.05. *n* = 3. (TIF 87 kb) Additional file 5: Figure S5. Western-blotting of TLR2 in purified astrocytes after TLZ treatment. (TIF 325 kb)

## Abbreviations

Ara C: Arabinoside C
BSA: Bovine serum albumin
CC3: Cleaved Caspase-3
CM: Conditioned medium
DMEM: Dulbecco’s modified eagle’s medium
dpi: Days post-injury
EM: Electronic microscopic
FBS: Fetal bovine serum
GdCl3: Gadolinium chloride
GDNF: Glial cell line-derived neurotrophic factor
GFAP: Glial fiber acid protein
HMGB1: High mobility group box protein 1
IDO: Indoleamine-2,3-dioxygenase
iNOS: Inducible nitric oxide synthase
LME: l-leucine methyl ester
LPS: Lipopolysaccharide
M-CSF: Macrophage colony-stimulating factor
MLKL: Mixed lineage kinase domain-like protein
MyD88: Myeloid differentiation primary response gene 88
Nec-1: Necrostatin-1
PBS: Phosphate buffered saline
PI: Propidium iodide
RIP1: Receptor-interacting protein 1
RIP3: Receptor-interacting protein 3
ROS: Reactive oxygen species
SCI: Spinal cord injury
TLR4: Toll-like receptor 4
TLZ: TNFα, LPS and z-VAD
TLZN: TLZ plus Nec-1
TNFα: Tumor necrosis factor alpha
