# Supplementary figures and images for: Reactive astrocytes undergo M1 microglia/macrohpages-induced necroptosis in spinal cord injury
Source: Mol Neurodegener. 2016 Feb 3;11:14. doi: 10.1186/s13024-016-0081-8 (PMC4740993; doi:10.1186/s13024-016-0081-8)

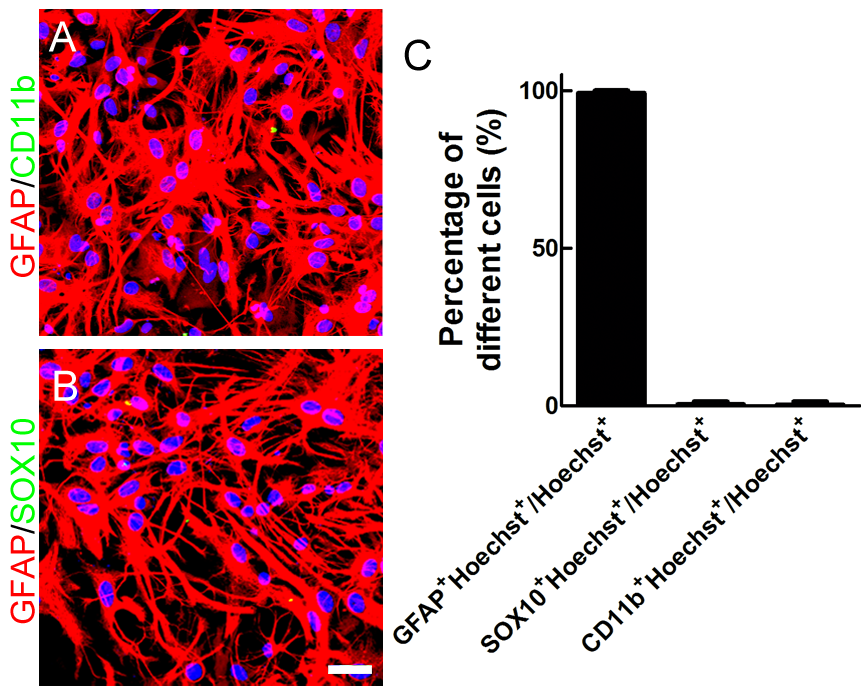

Supplement: Additional file 1: Figure S1. — Double-staining of GFAP with Sox10 and CD11b after astrocyte purification. Over 99 % of cells were GFAP-positive. Very rare could Sox10- and CD11b-positive cells be detected. Bar = 15 μm. (TIF 2385 kb) [file 13024_2016_81_MOESM1_ESM.tif]

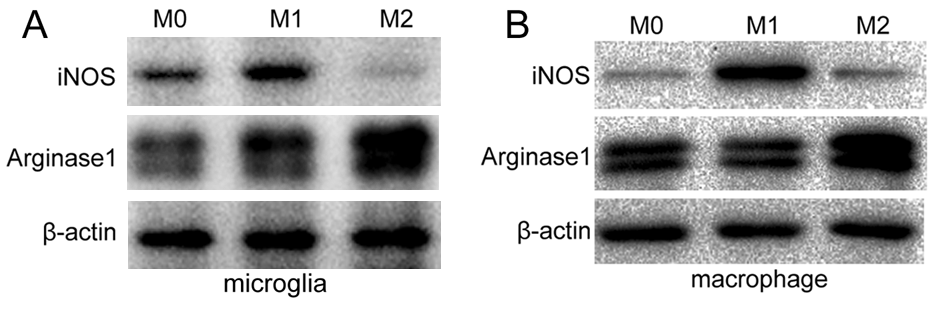

Supplement: Additional file 2: Figure S2. — Western-blotting of iNOS and Arginase 1 in M1 and M2 polarized micoglia and macrophages. (TIF 1374 kb) [file 13024_2016_81_MOESM2_ESM.tif]

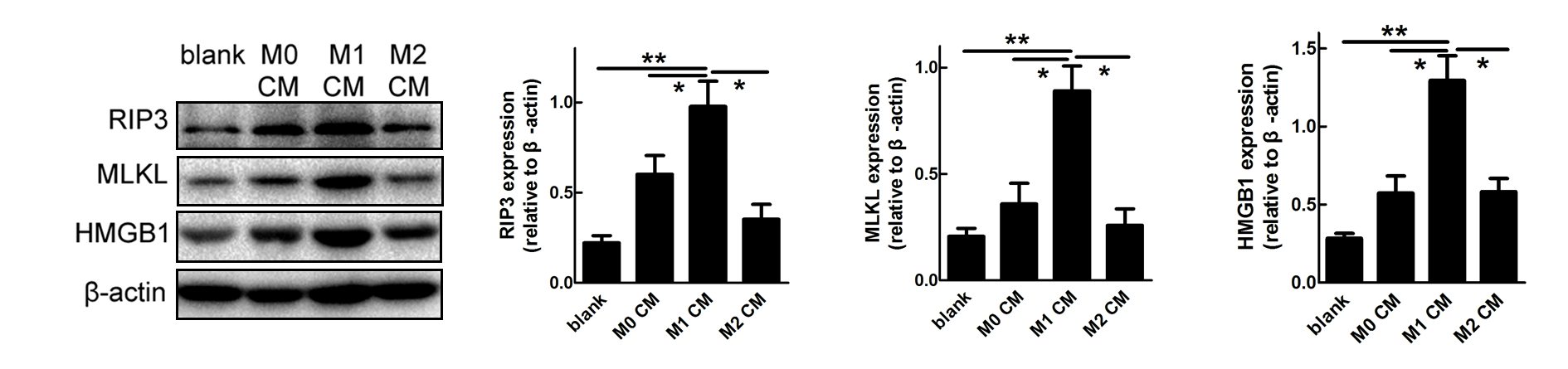

Supplement: Additional file 3: Figure S3. — Western-blotting and quantification of RIP3, MLKL and HMGB1 in astrocytes treated by conditioned medium from M0 microglia (M0 CM), M1 microglia (M1 CM), and M2 microglia (M2 CM). Notice that M1 CM has the strongest effects in stimulating the expression of RIP3, MLKL and HMGB1. *P <0.05, **P <0.01. n = 3. (TIF 3027 kb) [file 13024_2016_81_MOESM3_ESM.tif]

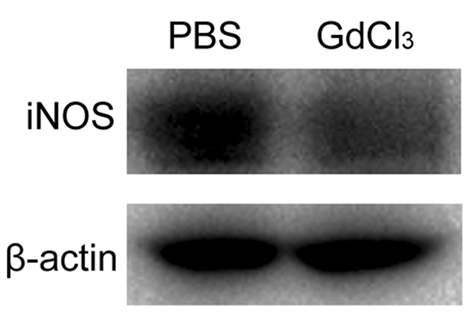

Supplement: Additional file 4: Figure S4. — Western-blotting of iNOS at 5 dpi in GdCl3 or PBS treated mice. The expression of iNOS was reduced in GdCl3 treated mice. *P <0.05. n = 3. (TIF 87 kb) [file 13024_2016_81_MOESM4_ESM.tif]

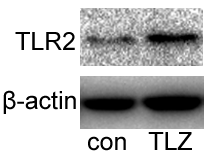

Supplement: Additional file 5: Figure S5. — Western-blotting of TLR2 in purified astrocytes after TLZ treatment. (TIF 325 kb) [file 13024_2016_81_MOESM5_ESM.tif]
